# Comparative Cytotoxic Effects and Possible Mechanisms of Deoxynivalenol, Zearalenone and T-2 Toxin Exposure to Porcine Leydig Cells In Vitro

**DOI:** 10.3390/toxins14020113

**Published:** 2022-02-02

**Authors:** Lingwei Sun, Jianjun Dai, Jiehuan Xu, Junhua Yang, Defu Zhang

**Affiliations:** 1 Institute of Animal Science and Veterinary Medicine, Shanghai Academy of Agricultural Sciences, Shanghai 201106, China; sunlingwei1987@126.com (L.S.); daijianjun@saas.sh.cn (J.D.); jiehuanxu810@163.com (J.X.); 2 Institute for Agri-Food Standard and Testing, Shanghai Academy of Agricultural Sciences, Shanghai 201403, China

**Keywords:** Leydig cell, zearalenone, deoxynivalenol, T-2 toxin, reproductive toxicity, in vitro

## Abstract

Mycotoxins such as zearalenone (ZEN), deoxynivalenol (DON) and T-2 toxin (T-2) are the most poisonous biological toxins in food pollution. Mycotoxin contaminations are a global health issue. The aim of the current study was to use porcine Leydig cells as a model to explore the toxic effects and underlying mechanisms of ZEN, DON and T-2. The 50% inhibitory concentration (IC_50_) of ZEN was 49.71 μM, and the IC_50_ values of DON and T-2 were 2.49 μM and 97.18 nM, respectively. Based on the values of IC_50_, ZEN, DON and T-2 exposure resulted in increased cell apoptosis, as well as disrupted mitochondria membrane potential and cell cycle distribution. The results also showed that ZEN and DON significantly reduced testosterone and progesterone secretion in Leydig cells, but T-2 only reduced testosterone secretion. Furthermore, the expression of steroidogenic acute regulatory (StAR) protein and 3β-hydroxysteroid dehydrogenase (3β-HSD) were significantly decreased by ZEN, DON and T-2; whereas the protein expression of cholesterol side-chain cleavage enzyme (CYP11A1) was only significantly decreased by ZEN. Altogether, these data suggest that the ZEN, DON and T-2 toxins resulted in reproductive toxicity involving the inhibition of steroidogenesis and cell proliferation, which contributes to the cellular apoptosis induced by mitochondrial injury in porcine Leydig cells.

## 1. Introduction

Leydig cells, situated in the testis interstitial, are responsible for the secretion of testosterone and other steroid hormones. They are also vital for the maintenance of secondary sexual characteristics and for promoting spermatogenesis in adult males [1]. Numerous environmental factors, including mycotoxins, have been shown to interfere with endocrine activity in Leydig cells. To date, an abundance of literature has been published on the cytotoxic effects of mycotoxins on Leydig cells in the rat [2,3] and mouse [4] as human models, but there is a dearth of research in swine. As an important model animal and an economic livestock species, the pig and its fertility have always received broad attention. The cell morphology and structure of porcine Leydig cells are very similar to those of humans; therefore, porcine Leydig cells could be chosen as an in vitro model for evaluating the potential endocrine-disrupting effect induced by mycotoxins [5,6].

Mycotoxins are produced as secondary metabolites by filamentous fungi that are present in many food commodities around the world [7]. It is known that agricultural commodities are susceptible to mycotoxin-producing fungi during harvest, transportation, processing, and storage. Mycotoxin contamination of raw materials, foods and feeds is a global threat to agricultural products’ safety and human health. The Food and Agriculture Organization (FAO) estimates that about 25% of world’s crops are affected by mycotoxins, and over five billion people are living with a high risk of exposure to these food contaminants [8]. Numerous in vitro studies have demonstrated that co-contamination of several mycotoxins could be characterized as synergistic, additive, or antagonistic [9]. However, there are limited and inconclusive data on combined toxic effects of mycotoxins due to differences in experimental designs and conditions. In order to better understand the mechanisms of toxin contamination, it is necessary to understand the mechanisms of single-toxin contamination.

Zearalenone (ZEN) and trichothecenes, including deoxynivalenol (DON) and T-2 toxin (T-2), are the most prevalent Fusarium mycotoxins worldwide, and have been recognized as important contaminants in food and animal feed [10]. These mycotoxins are harmful or toxic to animals and humans, among which swine are regarded as the most sensitive species to these toxins [11]. Although the amount of research on the influence of mycotoxins on hepatotoxicity, nephrotoxicity, neurotoxicity, and immunotoxicity has been rapidly increasing over the past few decades, limited data are available on mycotoxins’ effects on male reproductive health. It is of concern that the incidence of reproductive disorders, particularly in males, has been steadily increasing, and prior reports suggest that environmental exposure to the commonly encountered mycotoxins (DON, ZEN, T-2) may be related to this observation [12].

Notably, exposure to ZEN, DON and T-2 can reduce fecundity and decrease the expression of hydroxysteroid dehydrogenases (HSDs) responsible for sex steroids’ formation, including estrogen, progesterone, and testosterone [13]. Previous work has demonstrated ZEN exposure in mice causes testicular germ cell depletion, and influences steroidogenesis via the regulation of steroidogenesis-related key genes [4]. Several recent papers have demonstrated that ZEA induces apoptosis of mouse TM3 cells through the PI3K/AKT signaling pathway [14], and decreases steroidogenic gene expression in mouse primary LCs and the TM3 cell line, accompanied by diminished testosterone synthesis [15]. A recent review by Li et al. also showed that ZEN induces an imbalance of oxidative stress and estrogen, which contributes to reproductive injuries in rodents at all stages of their lives [16]. However, it is still unclear what the molecular mechanisms underlying reproductive alterations in pigs induced by ZEN exposure are. Similarly to ZEN, there have been many reproductive toxicity studies on DON and T-2 in mice or rats, and much less is known about how cells defend against the toxic effects with a focus on pigs [17,18].

Consequently, it is necessary to investigate the impact and associated mechanisms of ZEN, DON and T-2 on porcine Leydig cells’ steroidogenesis. In the present study, we used porcine Leydig cells as a reproductive model for the assessment of possible endocrine-disrupting effects of ZEN, DON and T-2 toxins, and aimed to identify their underlying mechanism. Firstly, we investigated the values of 50% inhibitory concentration (IC_50_) in Leydig cells after treatment with ZEN, DON and T-2 in various concentrations, and then the subsequent experiments were carried out using the IC_50_ values. In addition, we measured the effects of ZEN, DON and T-2 on the cell cycle, apoptosis, reactive oxygen species (ROS) levels, and the mitochondrial membrane potential (MMP) of Leydig cells using flow cytometry, respectively. Moreover, steroid hormone production in Leydig cells was determined. Finally, the effects of toxins on steroidogenesis-related proteins’ expression were investigated using Western blotting in Leydig cells.

## 2. Results

### 2.1. Effects of ZEN, DON and T-2 on Cell Proliferation

**Figure 1:**
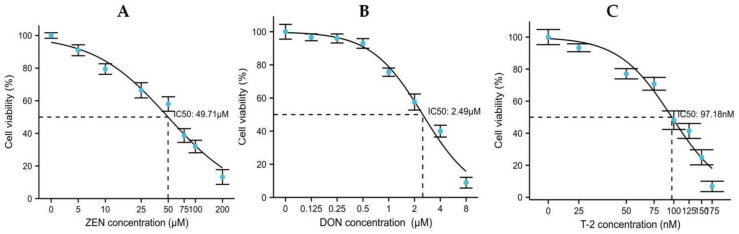
Cell viability of porcine Leydig cells after 24 h treatment with various concentrations of ZEN (Zearalenone; (**A**)), DON (deoxynivalenol; (**B**)), and T-2 (T-2 toxin; (**C**)) was evaluated by CCK-8 assay. Values were expressed as a percentage relative to 0.5% DMSO vehicle control.

Based on the data obtained from cell viability, the concentration–response curves for Leydig cells were established and IC_50_ values were evaluated (Figure 1). Our results reveal that ZEN, DON and T-2 decreased cellular viability in a concentration-dependent way compared to the vehicle control (0.5% DMSO). With T-2 treatment between 25 and 175 nM for 24 h, T-2 had the strongest cytotoxicity against Leydig cells (IC_50_: 97.18 nM). DON was the next most cytotoxic to Leydig cells with an IC_50_ value of 2.49 μM, and ZEN had the least cytotoxicity (IC_50_: 49.71 μM). In the follow-up experiment, cells were treated with 50 μM ZEA, 2.5 μM DON and 100 nM T-2, and harvested after 24 h treatment.

**Figure 2:**
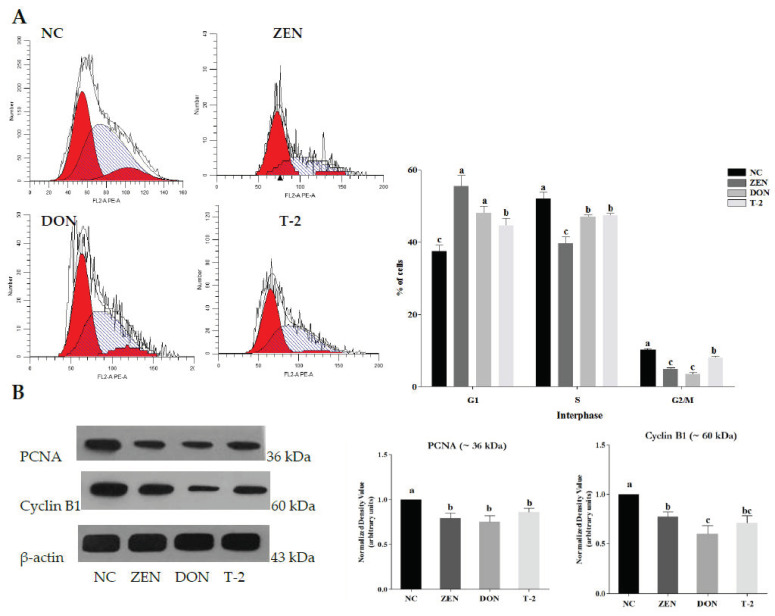
Effects of zearalenone (ZEN), deoxynivalenol (DON) and T-2 toxin (T-2) on cell cycle distribution in Leydig cells were analyzed by flow cytometry (**A**); protein expression of PCNA and cyclin B1 were detected using Western blotting (**B**); β-actin was detected as control. Values represent means ± SEM of triplicate results. Bar graph shows the relative expression of protein among the groups. The letters a, b, and c above the bars denote significant differences between groups (*p* < 0.05, one-way ANOVA).

Afterwards, the effects of ZEN, DON and T-2 on cell cycle distribution in porcine Leydig cells were further analyzed (Figure 2A). The results showed that the numbers of G1 phase Leydig cells after exposure to ZEN, DON and T-2 were amplified when compared with untreated controls, with a decreased number of cells in the S and G2/M phases (*p* < 0.05). These results indicated that ZEN, DON and T-2 may contribute to decreasing DNA synthesis in the cell cycle and inhibition of cell proliferation. To further explore the potential mechanisms, the protein expression of cell-cycle-related proliferating cell nuclear antigen (PCNA) and cyclin B1 in Leydig cells was examined. As shown in Figure 2B, the protein expression levels of PCNA and cyclin B1 were reduced significantly after treatment with ZEN, DON and T-2 (*p* < 0.05).

### 2.2. Effects of ZEN, DON and T-2 on Cell Apoptosis

**Figure 3:**
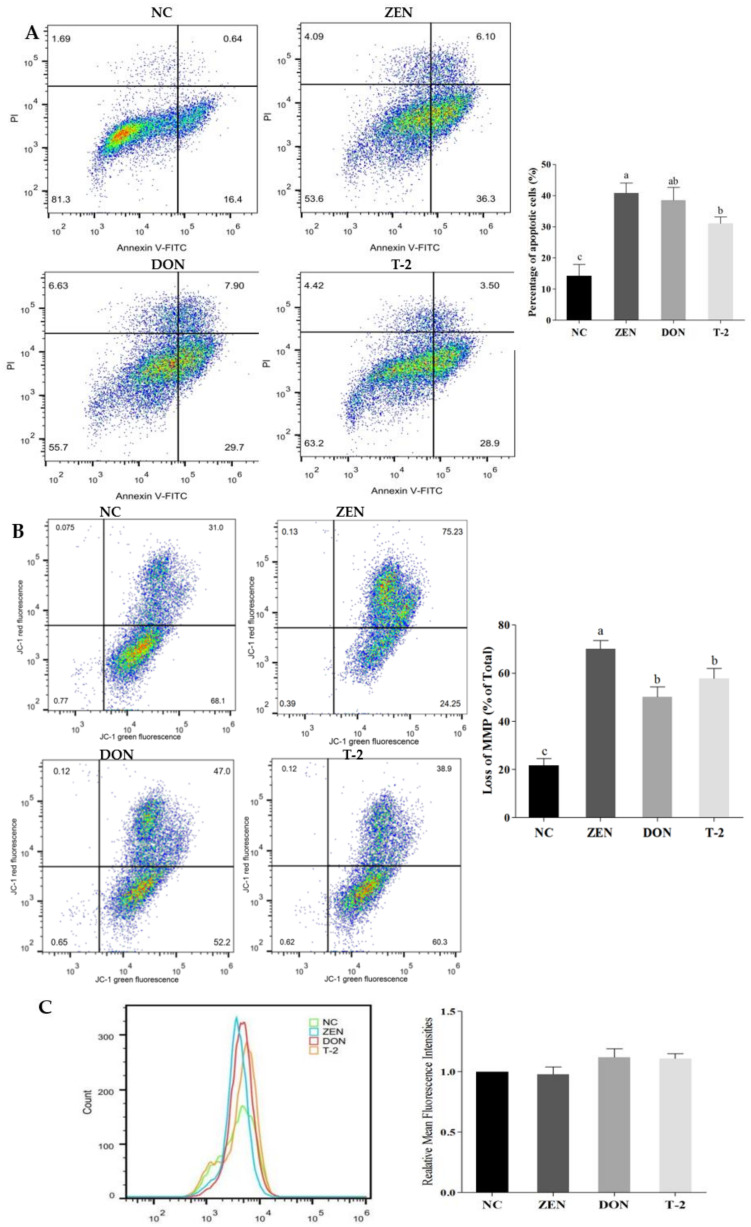
Effects of ZEN, DON and T-2 on the regulation of cell apoptosis, MMP and ROS levels in porcine Leydig cells. (**A**) The apoptotic cells were assessed by flow cytometry Annexin V-FITC/PI staining, (**B**) Changes in MMP were measured by JC-1 assay using flow cytometry. The loss of MMP was calculated by red/green fluorescence as 100%, (**C**) Intracellular ROS production was assessed by flow cytometry followed by DCFH-DA staining. Relative fluorescence intensity (RFI) of ROS is represented as the ratio relative to the control experiment. Values represent means ±  SEM of triplicate results. The letters a, b, and c above the bars denote significant differences between groups (*p* < 0.05, one-way ANOVA).

To assess the impact of ZEN, DON and T-2 on apoptosis, apoptosis of Leydig cells was analyzed by flow cytometry after Annexin V-FITC/PI staining. After ZEN, DON and T-2 treatment, apoptosis rates were considerably increased compared with the control group (Figure 3A, *p* < 0.05). This result indicated that cell apoptosis could be induced by ZEN, DON and T-2 in Leydig cells.

Disruption of MMP is an important signal of the early manifestation of apoptosis. In order to investigate whether mitochondria-mediated apoptosis is triggered, we assessed MMP using JC-1 staining. After treatment with ZEN, DON and T-2 in Leydig cells, the loss of MMP significantly increased compared with the controls (Figure 3B, *p* < 0.05).

Intracellular ROS production is an important factor affecting the induction of apoptosis and depolarizing the mitochondria. Therefore, we determined the relationship between apoptosis in cells following ZEN, DON and T-2 exposure and the changes in intracellular ROS levels. As shown in Figure 3C, intracellular ROS levels tended to increase after ZEN, DON or T-2 treatments, but not significantly (*p* > 0.05). As described above, no excessive ROS were produced in Leydig cells exposed to ZEN, DON and T-2, suggesting that mycotoxin-induced apoptosis was not due to ROS generation.

### 2.3. Effects of ZEN, DON and T-2 on Steroid Hormone Biosynthesis

**Figure 4:**
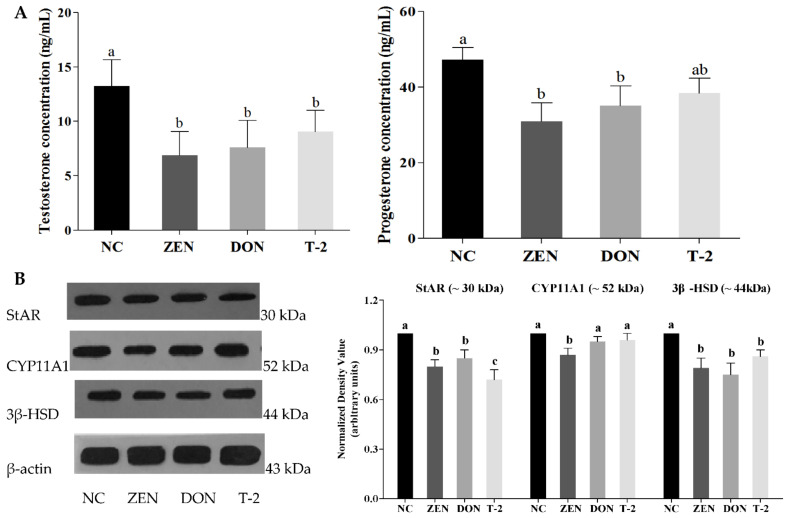
Effects of zearalenone (ZEN), deoxynivalenol (DON) and T-2 toxin (T-2) exposure on progesterone and testosterone secretion, and expression of key steroidogenic proteins in porcine Leydig cells. (**A**) Concentrations of progesterone and testosterone were determined using ELISA kits. (**B**) Protein expression of key proteins related to steroid hormone biosynthesis (StAR, CYP11A1 and 3β-HSD) were detected using Western blotting. β-actin was detected as control. Values represent means ±  SEM of triplicate results. The letters a, b, and c above the bars denote significant differences between groups (*p* < 0.05, one-way ANOVA).

To further validate the regulatory effect of ZEN, DON and T-2 on steroid hormone biosynthesis, steroid hormone levels and the expression of key proteins related to steroid hormone biosynthesis were analyzed. Firstly, we determined the concentration of steroid hormones including testosterone and progesterone by ELISA (Figure 4A). The results exhibited that ZEN, DON and T-2 could dramatically inhibit testosterone secretion in Leydig cells (*p* < 0.05). However, cells treated with ZEN and DON showed a significant reduction in progesterone secretion (*p* < 0.05), whereas progesterone secretion was not significantly changed by Leydig cells after exposure to T-2 (*p* > 0.05).

Subsequently, the expression of steroidogenic acute regulatory (StAR), cholesterol side-chain cleavage enzyme (CYP11A1), and 3β-hydroxysteroid dehydrogenase (3β-HSD) proteins were detected by Western blot. As shown in Figure 4B, the levels of StAR and 3β-HSD expression decreased significantly in the groups treated with ZEN, DON and T-2 compared with the control group (*p* < 0.05). Additionally, CYP11A1 protein expression was significantly decreased in the ZEN group (*p* < 0.05), but the difference was not significant between DON, T-2 and the control groups (*p* > 0.05).

## 3. Discussion

As one of the most important somatic cells, Leydig cells are very important for the maintenance of the male phenotype and spermatogenesis [1]. They are susceptible to mycotoxin contamination due to their high lipid content [19]. Mycotoxins, as a widespread natural contaminant, are the most poisonous biological toxins in food pollution [7]. Because of their high resemblance to human neonatal Leydig cells, porcine Leydig cells are an excellent model for studying potential endocrine disruption by mycotoxin exposure [6,19]. We used porcine Leydig cells in this study as an in vitro model to investigate the potential mechanisms and implications by which ZEN, DON and T-2 induce reproductive toxicity. More importantly, to our knowledge, this work is the first report on the mechanisms of ZEN, DON and T-2 in steroidogenesis in porcine Leydig cells.

In this study, we found that T-2 was the most cytotoxic in Leydig cells (IC_50_: 97.18 nM), followed by DON (IC_50_: 2.49 μM) and ZEN (IC_50_: 49.71 μM). Among mycotoxins produced by Fusarium species, T-2 is considered to be the most potent in terms of cytotoxicity [20]. This study is the first to assess the impact of different concentrations of ZEN, DON and T-2 on the viability of porcine Leydig cells. Wu et al. showed that cell viability of TM3 Leydig cells was approximately 50% after treatment with T-2 at a 100 nM concentration [21]. The IC_50_ value of DON was approximately 2.4 μM for renal proximal tubule epithelial cells after 24 h exposure, and the IC_50_ in Chinese hamster ovary K1 cells 48 h after treatment was 1.94 ± 0.52 μM [22]. In Caco-2 cells, the IC_50_ value was 49.5 µM for ZEN [23]. These IC_50_ values are in line with our results. However, cytotoxicity of DON and ZEN was found to be lower in MA-10 murine Leydig cells, with IC_50_ values of 0.25 µM and 34 µM, respectively [3]. A possible reason for this is the use of different species. Furthermore, ZEN, DON and T-2 exhibited a concentration-dependent inhibition effect on the cell reproductive system of males [3,24,25]. Another study showed that viability in Leydig cells, which was similar to that observed with low concentrations of ZEN (10 μM) exposure in murine Leydig cells, enhanced cell cycle progression and promoted proliferation, presumably due to the estrogenic potency of ZEN [3]. In this study, ZEN, DON and T-2 showed very different toxicity. Thus, for each toxicant, the subsequent experiments were chosen to approximate the IC_50_.

Then, ZEN-, DON- and T-2-suppressed cell growth was observed through controlling the cell cycle. Therefore, we used flow cytometry to evaluate cell cycle progression. The results showed that ZEN, DON and T-2 exerted a cytotoxic effect on Leydig cells through increasing cell cycles at the G1 phase and prevented transition from the G1 to the S phase. According to the literature, cell cycle arrest may be part of an adaptation procedure. In this process, DNA damage initiates the DNA damage checkpoint to guarantee proliferation of normal cells through the induction of cell cycle arrest [26]. Previous studies have also shown that the cells were able to arrest their multiplication cycle in the G1 phase for DNA repair and to maintain cellular homeostasis [27]. Interestingly, cell cycle analyses of rat Leydig cells showed cell cycle arrest at the S phase after treatment with various concentrations of dehydroepiandrosterone [28]. To probe the mechanism of cell cycle arrest further, cell-cycle-regulatory proteins PCNA and cyclin B1 were detected by Western blot analysis. This study showed that the expression levels of PCNA and cyclin B1 proteins were decreased in Leydig cells with ZEN, DON and T-2 exposure. During cell cycle progression, PCNA and cyclin B1 are involved in DNA repair, DNA replication and cell cycle regulation, and are expressed in the G1 late phase and S phase of the cellular cycle [29]. Furthermore, PCNA and cyclin B1 expressions display a positive correlation with germ cells’ proliferation [30]. As a result, the downregulation of protein expression levels of PCNA and cyclin B1 may be responsible for inhibiting the proliferation of Leydig cells, thereby inhibiting cell progression. The specific molecular mechanisms, however, remain to be elucidated.

The balance between DNA repair and cell apoptosis ultimately affects the cells’ activity, and cells are programmed to undergo apoptosis if the repair of the damaged DNA fails. As mentioned, mycotoxins can inhibit cell growth and influence cell apoptosis. Consistent with this, ZEN, DON and T-2 not only induced G1 phase cell cycle arrest, but also induced apoptosis in this study. This agrees with prior research demonstrating the capacity of ZEN and T-2 to trigger apoptosis in mouse Leydig cells [31,32]. Another study also demonstrated that DON can have a marked impact on cell apoptosis in granulosa cells [33]. Mitochondria, as core players in cellular energy metabolism, are involved in the apoptosis process. Hence, this study sought to elucidate the mitochondrial apoptotic pathway during apoptotic processes via measurement of MMP and ROS level. The results revealed that ZEN, DON and T-2 distinctly reduced MMP, thereby promoting cell apoptosis. A similar observation has been reported in the literature that ZEN treatment induces apoptosis in primary Leydig cells via decreased MMP expression. [32]. DON has also been known to cause apoptosis through the mitochondrial signaling pathway in a number of cell types [34]. A similar effect was observed in pig lymph node cells exposed to T-2 [35]. Therefore, it is suggested that mitochondrial dysfunction serves a critical role in ZEN-, DON- and T-2-mediated apoptosis in Leydig cells.

Synthesis and secretion of steroid hormones are the main functions of Leydig cells. Progesterone, as the precursor substance of various steroidal hormones such as testosterone, can regulate male fertility and testosterone biosynthesis [36]. Previous studies have revealed that ZEN and DON treatment of mouse Leydig cells had negative effects on progesterone and testosterone synthesis [3]. It has also been reported that T-2 has a variety of cytotoxic effects and has been proven to inhibit testosterone secretion [18]. As far as we know, the impacts of T-2 on the viability of progesterone synthesis in Leydig cells has never been reported. In the present study, it was observed that exposure to ZEN, DON and T-2 toxins inhibited testosterone synthesis in Leydig cells, but the progesterone level was only reduced in ZEN- and DON-treated groups. These results suggest that ZEN and DON may negatively influence Leydig cell testosterone synthesis by inhibiting the conversion of progesterone into testosterone. The present results also demonstrated that the impacts of T-2 on testosterone synthesis are not mediated by progesterone.

Steroid hormone biosynthesis in Leydig cells may be associated with rate-limiting enzymes in the steroid biosynthesis pathway. These include some important proteins, such as steroidogenic acute regulatory (StAR), cytochrome P450 side-chain cleavage enzyme (CYP11A1), and 3β-hydroxysteroid dehydrogenase (3β-HSD), which play crucial roles in anabolic pathways [37]. Given the importance of these proteins in facilitating steroidogenesis, the expression levels of StAR, CYP11A1 and 3β-HSD proteins in Leydig cells were also assessed. We found that ZEN, DON and T-2 decreased the expression of StAR and 3β-HSD, but only ZEN reduced the expression of CYP11A1. The decreased protein expression in ZEN-, DON- and T-2-exposed Leydig cells was also accompanied by reduced activities of steroidogenic enzymes, which suggests a possible direct inhibitory effect of mycotoxins on steroidogenic enzymes’ expression. Similar results have previously been published by other workers. It has been previously reported by Yang et al. that T-2 attenuates testosterone secretion via regulating the activities of CYP11A1, 3β-HSD and StAR in mouse Leydig cells [38]. Liu et al. also demonstrated downregulation of the expression of steroidogenic enzymes in mouse Leydig cells, as well as decreased mRNA levels of StAR, CYP11A1 and 3β-HSD after ZEN treatment. These results indicated that ZEN, DON and T-2 may interrupt steroid biosynthesis in vitro by disturbing expression of genes or proteins associated with steroid biosynthesis.

## 4. Conclusions

In conclusion, this study showed that ZEN, DON and T-2 inhibit porcine Leydig cell proliferation, primarily by inducing cell death via apoptosis, and possibly also via the induction of ROS and by disrupting MMP and cell cycle distribution. This study also demonstrates that ZEN, DON and T-2 inhibit steroidogenesis by decreasing steroidogenic enzymes. This work provides novel insights to guide further studies on this species. Thus, it will be worthwhile to further validate these findings in more extensive studies.

## 5. Materials and Methods

### 5.1. Materials

The mycotoxins ZEN, DON, and T-2 were obtained from Sigma Chemical Co. (St. Louis, MO, USA). All mycotoxins were dissolved in dimethylsulfoxide (DMSO). DMEM and fetal bovine serum (FBS) were obtained from Gibco-Life Technology (Eggenstein-Leopoldshafen, Germany). The final concentration of DMSO in each culture was 0.5%, and controls received vehicle (0.5% DMSO) alone.

### 5.2. Animals

The experimental studies were strictly performed in accordance with the guidelines of the regional Animal Ethics Committee for experimental animals, Shanghai Academy of Agricultural Sciences, China (protocol code SAASPZ0920001. Approval Date: 5 January 2021).

### 5.3. Isolation and Culture of Porcine Leydig Cell

Leydig cells were isolated from porcine testes (15–20 days of age) as previously described in Salva et al. [39] Briefly, testes were decapsulated and digested in dissociation buffer (ice-cold Ham’s F12 and DMEM 1:1 supplemented with sodium bicarbonate (1.2 mg/mL), gentamycin (16 g/mL), 1% penicillin/streptomycin, 0.1% nystatin, fortum (8 mg/mL) and Hepes (15 mM); DMEM/F12). Following removal of fibrous capsule, the testes were minced and homogenized in DMEM/F12 containing 0.5 mg/mL collagenase at 34 °C for 90 min. Cell suspension was filtered through a 40 mm nylon mesh, and then collected by centrifugation at 250 g for 10 min. The cells were resuspended in DMEM/F12 medium. After sedimentation twice at unit gravity, the suspensions were purified by Percoll gradient separation and centrifuged at 800 g for 20 min. Freshly isolated Leydig cells were suspended in DMEM/F12 containing 10% FBS and 1% penicillin/streptomycin, and plated in six-well culture plates (2.5 × 10^6^ cells/well) and cultured at 37 °C, under 5% CO_2_.

The purity of Leydig cells was identified by staining for 3β-hydroxysteroid dehydrogenase (3β-HSD), as described in reference [40]. Leydig cell purity was more than 95%. Cells were subcultured every 2–3 days at a ratio of 1:3 to maintain their exponential growth phase, for a maximum of 12 passages. Cells were plated in 25 cm^2^ flasks and cultured for 24 h to allow for cell attachment. Medium was changed once every 2–3 days until confluent monolayers were formed. Thereafter, the cultured cells were either subcultured or used for experiments.

### 5.4. Cell Treatment

After reaching about 80% confluence, the medium was replaced by DMEM/F12 containing various concentrations of DON, ZEN, or T-2 for 24 h (showed in Table S1). Controls were inoculated with blank culture medium.

### 5.5. Cell Proliferation Assay

Cell counting Kit-8 (CCK-8; Beyotime Biotechnology, Haimen, China) was used to measure cell viability according to the manufacture’s protocol. First, cell suspensions were seeded in 96-well plates (3000 cells/well). When grown to 80% confluence, cells were treated with DON, ZEN, or T-2 at different concentrations in 5% CO_2_ at 37 °C for 24 h. Subsequently, cells were incubated with 100 μL of fresh DMEM containing 10% CCK-8 reagent at 37 °C for 3 h. Absorbance readings were recorded at 450 nm using a microculture plate reader (BioTek, Winooski, VT, USA). This assay was independently performed three times, with four replications each time. Cell viability (%) was calculated as sample absorbance/control absorbance ×100%. All the concentration response curves and the IC50 values were analyzed using GraphPad Prism software (version 4.0, GraphPad Software, San Diego, CA, USA).

### 5.6. Cell Cycle Assay

The cell cycle was assessed by flow cytometry using a cell cycle analysis kit (Multi Sciences, Hangzhou, China). After 24 h of treatment, cells were harvested, washed in PBS, and fixed with 75% ice-cold ethanol overnight at 4 °C. After two PBS rinses, cells were counterstained in 500 μL of PBS containing propidium iodide (PI, 40 μL/mL) and RNase (100 μL/mL) for 30 min at room temperature in the dark. Cell cycle analysis was assessed based on DNA content using flow cytometer (FACS Calibur) (Becton Dickinson, San Jose, CA, USA). Data were analyzed by the ModFit LT software packages (version 3.0, Verity Software House, Inc., Topsham, ME).

### 5.7. Cell Apoptosis Assay

Cell apoptosis was determined by the Annexin V-FITC/PI kit (Thermo Fisher Scientific, Waltham, MA, USA). After treatment, cells were harvested and washed in PBS, then resuspended in 96 μL annexin V-binding buffer (140 mM NaCl, 10 mM HEPES, 2.5 mM CaCI_2_, pH 7.4). Then, cells were stained with 1 μL FITC annexin V and 12.5 μL propidium iodine (10 μg/ mL) for 15 min at room temperature in the dark. Approximately 10,000 cells were collected for flow cytometry assay using a flow cytometer with excitation and emission at 488 and 530 nm, respectively.

### 5.8. MMP Assay

MMP was monitored using the JC1-mitochondrial membrane potential assay kit (Abcam; Cambridge, MA, USA), according to the manufacturer’s instructions. After 24 h treatment, cells in six-well plates were washed in PBS and harvested. JC-1 was then added to 1× dilution buffer to a final concentration of 10 μM. After 20 min incubation in the dark, the cells were washed twice with 1× dilution buffer. Then, cells were washed with assay buffer and acquired immediately by the flow cytometer. In normal cells with high mitochondrial membrane potential, JC-1 accumulates in the mitochondria and they fluoresce red. In damaged cells, JC-1 remains in the cytoplasm as green monomers. Finally, the MMP levels were calculated by the red-to-green fluorescence ratio.

### 5.9. ROS Production Assay

Cellular ROS levels were determined using a 2,7-dichlorofluorescin-diacetate (DCFH-DA; Beyotime, Haimen, China) probe. Intracellular DCFH-DA is cleaved by esterases to produce 2′,7′-dichlorodihydrofluorescein, which can be oxidized to the fluorescent compound 2′,7′-dichlorodihydrofluorescein in the presence of ROS. After being subjected to the treatments, samples were washed using PBS twice, and cells (2 × 10^4^ cells) were stained with DCFH-DA (10 μM) at 37 °C for 20 min. Then, cells were washed with PBS and replaced with 100 µL fresh phenol-red-free RPMI media. Fluorescence was monitored by flow cytometry. Results are shown as a percentage of the value of control.

### 5.10. Steroid Hormone Quantifications

Testosterone and progesterone concentrations in the supernatant were assayed using specific radioimmunoassay (RIA) kits (Diagnostic Products Inc., Los Angeles, CA, USA). All measurements were carried out according to the manufacturer’s instructions, and standard curves were prepared in the experimental cell culture medium. Assay sensitivity limits were 0.03 ng/mL for testosterone and 0.35 ng/mL for progesterone. According to the standard curves, the ranges of detection for the testosterone and progesterone concentrations were 0.12–36 ng/mL and 0–100 ng/mL, respectively. Inter- and intra-assay coefficients of variation were less than 10% for all treatments. Testosterone and progesterone concentrations are expressed as nanograms (ng) per 10^6^ cells from three independent experiments.

### 5.11. Western Blot Analysis

Leydig cells, including those treated with 50 μM ZEA, 2.5 μM DON and 100 nM T-2, were harvested 24 h after treatment. Then, samples were lysed on ice using radioimmunoprecipitation (RIPA) buffer (Solarbio, Beijing, China) for 30 min. A bicinchoninic acid kit (BCA; Pierce Chemical Co., Rockford, Illinois, USA) was used to evaluate the total protein concentration according to the manufacturer’s protocol. A total of 20 μg protein was electrophoresed on 10% SDS-PAGE and transferred onto nitrocellulose membrane (Bio-Rad, Hercules, CA, USA). After blocking with 5% skimmed milk for 1 h, membranes were incubated with primary antibodies overnight. The antibodies used in this study were as follows: anti-StAR (bs-20387R, dilution 1:1000) (Bioss, Beijing, China), anti-CYP11A1 (sc-18043, dilution 1:1000) (Santa Cruz, CA, USA), anti-3β-HSD (AT0816, dilution 1:1000) (CMCTAG, Dover, DE, USA), anti-cyclin B1 (AT0815, dilution 1:1000) (CMCTAG, Dover, DE, USA), anti-PCNA (AT1577, dilution 1:2000) (CMCTAG, Dover, DE, USA), and anti-β-actin (HC201, dilution 1:5000) (TransGen Biotech, Beijing, China). Afterwards, the membranes were incubated with the corresponding secondary antibody. Quantitative analysis of protein band intensities was performed with imaging software (ImageJ, 1.47q, National Institutes of Health, Bethesda, MD, USA).

### 5.12. Statistics

All data were analyzed using SPSS statistical software (version 20.0, SPSS Inc., Chicago, IL, USA). Data normality was assessed via the Shapiro–Wilk Test. Within-group differences were assessed by one-way ANOVA with post-hoc Bonferroni tests. *p*-values < 0.05 were regarded as statistically significant. Values are presented as means ± standard errors (SEM).

## Data Availability

Not applicable.

## Supplementary Materials

The following supporting information can be downloaded at: https://www.mdpi.com/article/10.3390/toxins14020113/s1, Table S1: Mycotoxin concentrations used in the presented study.
