# Supplementary material for: Comparative Cytotoxic Effects and Possible Mechanisms of Deoxynivalenol, Zearalenone and T-2 Toxin Exposure to Porcine Leydig Cells In Vitro
Source: Toxins (Basel). 2022 Feb 2;14(2):113. doi: 10.3390/toxins14020113 (PMC8875536; doi:10.3390/toxins14020113)
Supplement: Supplementary file 1 [file toxins-14-00113-s001.zip › toxins-1553782-supplementary.pdf]

Table S1. Mycotoxins concentrations used in presented study.

Toxins	Concentrations	Concentrations (ng/mL)
ZEN	5 μM	1591.8
	10 μM	3183.6
	25 μM	7959
	50 μM	15918
	75 μM	11938.5
	100 μM	31836
	200 μM	63672
DON	0.125 μM	37.04
	0.25 μM	74.08
	0.5 μM	148.16
	1 μM	296.32
	2 μM	592.64
	4 μM	1185.28
	8 μM	2370.56
T-2	25 nM	11.66
	50 nM	23.33
	75 nM	34.99
	100 nM	46.65
	125 nM	58.31
	150 nM	69.98
	175 nM	81.64

Abbreviations. ZEN: zearalenone; DON: deoxynivalenol; T-2: T-2 toxin.
